# King's College Hospital Revisited

**Published:** 1888-10-27

**Authors:** 


### KING'S COLLEGE HOSPITAL REVISITED.

*(By an Occasional Correspondent.)*

I HAD occasion the other day to call at King's College Hospital, and while there I was kindly asked by the sister matron if I would care to look over the hospital. It being some years since I had been in any of the wards, and knowing that changes had lately been made in the nursing, I gladly availed myself of the offer. I was first shown into one of the long wards devoted to female medical cases, where I was struck by the order, cleanliness, and general comfort of all I saw around me. The walls were tastefully painted in two shades of blue, with which the red and white counterpanes on the beds exactly harmonised. The nurses in their light, bright uniforms, the flowers on the tables, the pictures on the walls were all in accord with the repose so needful in a sick ward. Further, a few words with the sisters and nurses convinced me that their hearts were in the work they had set their hands to. We then visited other wards, the appearance of which showed the same care and attention had been bestowed upon them. The children's ward is delightful; it has a southern aspect, there is plenty of sun and light, and all round the room kind hands had painted such pictures as children love. Passing on to the nurses quarters, in which, perhaps, at that moment I was most interested, I found them situated at the top of the building, and are for the most part quite new. The arrangements are all that can be desired by the most critical, and when a nurse enters here she may feel perfectly at home. When a King's College nurse is off duty she is very properly left to do as she will—the restraint, care, and discipline of the ward being laid aside, and every comfort and convenience placed at her disposal. A carpeted apartment, with sofa and easy chairs, is provided for the general sitting-room of the nurses. A similar room, but somewhat smaller, is devoted to the probationers, and a very bright recreation hall, hung with some excellent proof engravings (the gift of a friend), is common to both nurses and probationers. A lecture theatre adjoins, where instruction and demonstrations are given at stated times by members of the medical staff; a piano also is provided for those who care to play or sing, and no reasonable amusement or diversion is prohibited. Nothing is neglected for the comfort of the nursing staff, provision being even made against colds and minor ailments by a constant supply of simple remedies which are kept in the Home. Every probationer has a little room to herself, neatly furnished with every necessary, and each nurse has her own cubicle, which is fitted with the same care and attention to detail. The air of comfort and homeliness about this department is greater than in any hospital I have seen, and I was not surprised when I was told that the applications for admission as probationers is far in excess of the number of vacancies. I further understood that for purposes of recreation every nurse can go out for a short time each day and during the day time, so that no nurse is compelled either to walk about in the night or to forego fresh air, which is necessary above all things to keep a nurse healthy.

No words of ours can so eloquently testify to the efficient administration of the nursing department at King's College Hospital at the present time, as the circumstantial account of the arrangements here given. Indeed, the Committee and Governors are to be congratulated on obtaining the services of so efficient and capable a lady as the present sister matron.

